# Presence of Exogenous Sulfate Is Mandatory for Tip Growth in the Brown Alga *Ectocarpus subulatus*

**DOI:** 10.3389/fpls.2020.01277

**Published:** 2020-08-18

**Authors:** Amandine Siméon, Sonia Kridi, Bernard Kloareg, Cécile Hervé

**Affiliations:** Sorbonne Universités, CNRS, Integrative Biology of Marine Models (LBI2M), Station Biologique de Roscoff, Roscoff, France

**Keywords:** tip growth, sulfated fucans, sulfate, extracellular matrix, cell wall, brown algae, *Ectocarpus*, alginate

## Abstract

Brown algae (Phaeophyceae) are multicellular photoautrophic organisms and the largest biomass producers in coastal regions. A variety of observations indicate that their extracellular matrix (ECM) is involved with screening of salts, development, cell fate selection, and defense responses. It is likely that these functionalities are related to its constitutive structures. The major components of the ECM of brown algae are β-glucans, alginates, and fucose-containing sulfated polysaccharides. The genus *Ectocarpus* comprises a wide range of species that have adapted to different environments, including isolates of *Ectocarpus subulatus*, a species highly resistant to low salinity. Previous studies on a freshwater strain of *E. subulatus* indicated that the sulfate remodeling of fucans is related to the external salt concentration. Here we show that the sulfate content of the surrounding medium is a key parameter influencing both the patterning of the alga and the occurrence of the BAM4 sulfated fucan epitope in walls of apical cells. These results indicate that sulfate uptake and incorporation in the sulfated fucans from apical cells is an essential parameter to sustain tip growth, and we discuss its influence on the architectural plasticity of *Ectocarpus*.

## Introduction

The molecular mechanisms determining apical growth have been studied in details in fungi and land plants (i.e., pollen tube, root hairs, moss protenemata). In fungal hyphae and land plant model systems, the cell wall is one of the structural key players regulating the shape and the heterotrophic growth of apical cells (Meekes, 1986; Lee et al., 2005; Parre and Geitmann, 2005; Riquelme et al., 2011). In pollen tubes, pectins and callose are abundant cell wall polymers. Yet the tip is devoid of callose and the degree of pectin methyl-esterification from tip-to-flank correlates with an increase in the degree of cell wall rigidity and a decrease in viscoelasticity. This affects extensibility of the cell wall and thus growth of the pollen tube (Parre and Geitmann, 2005; Chebli et al., 2012).

Tip growth has also been investigated in brown algae, either in filaments (e.g., Ectocarpales, Sphacelariales) or in more complex structures at apical meristems (e.g., Dictyotales), or during specific developmental stages, e.g., the rhizoid cell of *Fucus* embryos (Katsaros, 1995; Charrier et al., 2012). The zygote of various *Fucus* species has long served as a model to study molecular mechanisms during early embryogenesis and the initiation of the main body axis (Quatrano and Stevens, 1976; Bisgrove and Kropf, 2001; Bisgrove and Kropf, 2008). In *Fucus* zygotes, the first asymmetrical cell division defines the apical and basal cell lineages, named the thallus and rhizoid cells, respectively. Brown algal cell-walls are made of fucose-containing sulfated polysaccharides (FCSPs) interlocking a cellulosic scaffold and embedded within an alginate-phenol network (Deniaud-Bouët et al., 2014). In *Fucus* early embryogenesis, several studies have demonstrated the essential role of the cell-wall as a source of position-dependent information required for cell polarization. Cellulose and alginates are the first polysaccharides to be deposited uniformly into the wall after fertilization. The FCSPs are deposited at a later stage, during the establishment of polar axis and specifically at the emerging, rhizoid tip (Quatrano and Stevens, 1976; Bisgrove and Kropf, 2001). These observations were confirmed in *Fucus serratus* with monoclonal antibodies directed toward alginates and sulfated fucans (Torode et al., 2016).

These properties of the embryos of Fucales make them an excellent experimental model to study the cell biology and biochemistry of tip growth and cell polarity at the early stages of development. However, later stages of *Fucus* are not amenable to culture, in contrast to species from the Ectocarpales order, from which genomic resources are also available. Thus, Ectocarpales are emerging as a complementary model to Fucales to unravel the molecular bases of polar organization and multicellular development in brown algae, including the function of extracellular matrix polysaccharides. During the early stages of development of Ectocarpales, growth of the primary filaments is driven by the elongation and the division of the polarized, apical cells. Subsequent axillary branching (secondary filaments) emerge preferentially in the central core of the primary filaments and initiate new regions of apical growth (Charrier et al., 2008; Le Bail et al., 2008). Since the completion of the genome of the marine *Ectocarpus* sp,. Ec32 strain, formerly described as *E. siliculosus* (Cock et al., 2010), and the development of various genetic and genomic tools in this model brown alga (Cock et al., 2014; Tarver et al., 2015; Avia et al., 2017), they provide additional opportunities to investigate the biochemical and, potentially, the genetic bases of tip growth.

The genome of *E. subulatus*, Bft15b strain, a species which is able to acclimate to low salinity, has recently been sequenced (Dittami et al., 2020a). Only minor differences in the number of cell-wall genes, including encoded sulfatases and sulfotransferases, were identified as compared to the marine reference *Ectocarpus* sp., Ec32 strain. This indicates that the low salinity level of *E. subulatus* natural environment, as compared to marine salinity, did not drive a loss of the sulfatases/sulfotransferases genes. Primary growth and branching activity of *E. subulatus* Bft15b are influenced by the salinity of the surrounding medium (**Supplementary Figure S1**). These morphological variations are similar to those observed in a closely related *E. subulatus*, Ec371 strain, in which they correlated with changes in the expression of carbohydrate sulfotransferases and sulfatases (Dittami et al., 2012). In particular the expression of a sulfotransferase involved in the sulfation of sulfated fucans was increased by 32-fold when this *Ectocarpus* isolate was acclimated from diluted to normal seawater (Dittami et al., 2012). Conversely the acclimation of this isolate to higher salinity was accompanied by marked changes in the composition of the ECM. As shown by immunofluorescence imaging, the BAM4 monoclonal antibody, which is specific of highly sulfated fucans, was not detected at highly diluted seawater (5% of normal seawater), but conspicuous staining with BAM4 was observed upon culture of *E. subulatus* filaments in full-strength seawater (Torode et al., 2015).

In this study, we investigated the morphology of *E. subulatus*, Bft15 strain, and the incorporation of sulfated fucans in the extracellular matrix of growing filaments in function of the ionic strength and the sulfate content of the culture medium. We confirm that the branching activity of *E. subulatus* filaments depends on the salinity of seawater. However, it is the presence of sulfate in the culture medium, and not its overall ionic strength, which triggers growth of the primary filaments as well at the emergence of secondary filaments. We show that this developmental pattern correlates with the incorporation of sulfated fucans at the dome of apical cells, and we discuss the underlying mechanisms likely to affect the viscoelasticity of the extracellular matrix.

## Materials and Methods

### Algal Material

Two *Ectocarpus* strains (Ectocarpales, Phaeophyceae) were used: the *Ectocarpus* sp. Ec32 marine strain (Culture Collection of Algae and Protozoa accession no. 1310/4; origin San Juan de Marcona, Peru) used as a reference (Cock et al., 2010) and the *E. subulatus* strain Bft15b (Culture Collection of Algae and Protozoa CCAP accession 1310/34), isolated in 1978 by Dieter G. Müller in Beaufort, North Carolina, USA, and characterized by a high abiotic stress tolerance (Dittami et al., 2020a). All experiments were carried out in Petri dishes using unialgal laboratory cultures of haploid parthenosporophytes. The latest were produced by germination of unfertilized gametes. To do so, gametophytes featuring mature gametangia were placed in the dark in a small amount of seawater for 4 h (simulating low tide). The synchronized release of the gametes was induced by the addition of Provasoli-enriched seawater and the application of high light (intensity of 40 µmol m^−2^ s^−1^; simulating high tide). Gametes were separated from the parents by pipetting.

### Culture Conditions

Once released, the gametes were cultivated in a controlled-environment cabinet at 13°C with a photoperiod of 14-h light and 10-h darkness. During the first 24 h the gametes were allowed to settle down in a limited volume of either natural (NSW) or artificial seawater (ASW; 8.85 mM KCl, 0.55 M NaCl, 10 mM CaCl_2_, 16 mM MgSO_4_, 51.6 mM MgCl_2_, 1.77 mM NaHCO_3_, pH 7.8), before full immersion in the targeted medium. To assay tolerance to various salinities, cultures were grown in either undiluted (100%) or diluted (10%) seawater. Two *E. subulatus* strains, Bft15b (present study) and Ec371 (Dittami et al., 2012), are known to generate distinct morphotypes upon salinity variations, suggesting that such morphological changes may constitute a selective advantage during colonization of habitats (Dittami et al., 2012; Dittami et al., 2020a; Dittami et al., 2020b).

Past studies have shown that cultivating *Fucus* embryos in sulfate-depleted media complemented with ^35^S-methionine did not inhibit protein synthesis and that the sulfur of methionine was not utilized in sulfating fucans in the absence of sulfate (Crayton et al., 1974). To assay responses to sulfate starvation, cultures were grown in ASW where the sulfate was deleted and replaced by 10 mM methionine to preserve protein synthesis. To assay the reversibility of sulfate addition or depletion, cultivation in sulfate-free or normal ASW was initiated and the medium changed accordingly after 3 days then maintained up to 15 days of culture. In all cases the medium was autoclaved prior to use and subsequently enriched in Provasoli nutrients (Coelho et al., 2012), but featuring no sulfate. For morphogenesis observations the parthenosporophytes were grown in 55-mm Petri dishes, while sterile microscopic slides in 140 mm-Petri dishes were used for immunolabeling experiments.

### Growth and Morphogenesis Observations

Detailed observations were made on parthenosporophytes after 7, 10 and 15 days of cultivation following the gamete release. The number of cells within primary filaments (PFs) and the number of secondary filaments (SFs) were scored on at least four biological replicates and two technical replicates for each time point and each culture condition. These observations were performed on an Olympus IX 51 inverted microscope. Statistical analyses were performed using the R software. Shapiro-Wilk normality test assessments were made followed by the use of Mann-Whitney tests.

### Immunolabeling and Fluorescence Microscopy

Parthenosporophytes were fixed for 1 h in seawater containing 4% paraformaldehyde and 10% glycerol, washed twice in PBS and blocked with 5% (w/v) milk powder in PBS (PBS/MP) for 1 h. Samples were then washed in PBS. Fluorescence imaging of sulfated fucans and alginates was achieved with the use of the BAM4 antibody (Torode et al., 2015) and the BAM6 (M-rich alginate) and BAM7 (MG-alginates) antibodies (Torode et al., 2016), respectively. A 10-fold dilution of the BAM hybridoma supernatants was added in PBS/MP and incubated for 1 h. Following washing with PBS, the secondary anti-rat antibody linked to fluorescein isothiocyanate (FITC) was added at 100-fold dilutions in PBS/MP in darkness. Corresponding negative control samples were labeled with the secondary antibody only. In addition to the BAM antibodies, the binding of three further monoclonal antibodies were screened in this study using indirect immunofluorescence: LM15, a rat monoclonal antibody to xyloglucan (Marcus et al., 2008), a kind gift from Prof. Paul Knox (University of Leeds, UK), BS-400-2 (BioSupplies), a mouse monoclonal antibody to (1→3)-β-d-glucan (Meikle et al., 1991) and BS-400-3 (BioSupplies), a mouse monoclonal antibody to (1→3),(1→4)-β-d-glucan. The immunolabeling procedure was performed as stated above with the use of appropriate secondary antibodies (e.g., anti-rat linked to FITC for LM15 and anti-mouse linked to FITC for BS-400-2 and BS-400-3, respectively; Sigma). After 1 h, the samples were washed with PBS, incubated with 0.25% Calcofluor White (Sigma) for 5 min in darkness and mounted after washing in an anti-fading solution (Citifluor AF3; Agar Scientific). Omitting the primary antibody from the immunolabeling procedure on equivalent samples resulted in no observed fluorescence (**Supplementary Figure S2**). Calcofluor White stains β-glucans and cellulose in this case, as confirmed by a 0.1% solophenyl flavine 7GFE (Sigma) staining of equivalent filaments (**Supplementary Figure S3**). All slides were observed with an Olympus BX60 microscope equipped with epifluorescence irradiation. Images were captured with an Exi Aqua camera (Qimaging) and the Volocity software (Perkin Elmer).

## Results

### Cultivation of *E. subulatus* in Natural vs Diluted Seawater Promotes Filament Branching

**Figure 1:**
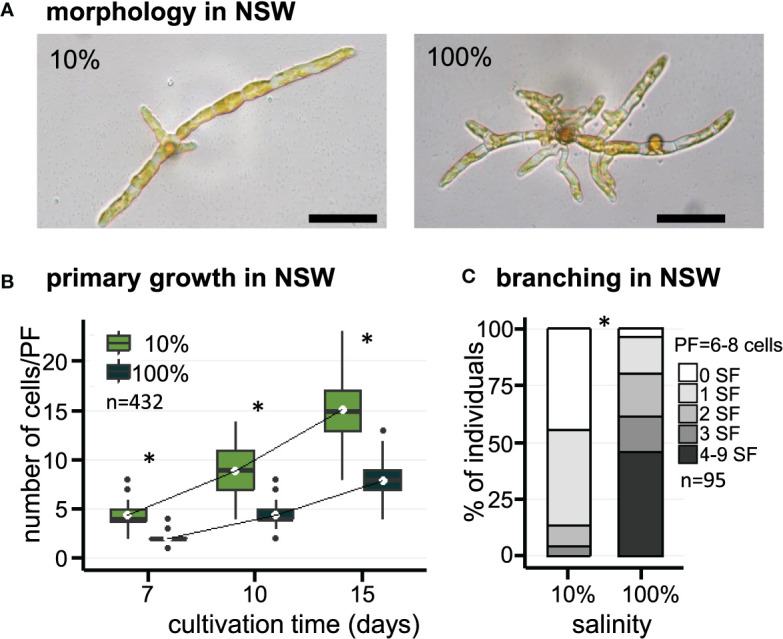
Morphology of *E. subulatus* grown in natural seawater (NSW) featuring distinct salinities. **(A)** Morphology of *E. subulatus* parthenosporphytes cultivated for 15 days in NSW featuring distinct salinities. Scale bar = 50 µm. **(B)** Elongation of *E. subulatus* primary filament (PF) was significantly greater (Mann-Whitney test: p < 0.001) when the gametes were cultivated in diluted seawater (10%) rather than in undiluted seawater (100%). **(C)** The branching activity of *E. subulatus* was significantly greater (Mann-Whitney test: p < 0.001) when the gametes were cultivated in undiluted seawater (100%) rather than in diluted seawater (10%). * above bars indicates significant differences.

The effect of salinity on the early morphogenesis of *E. subulatus*, strain Bft15b, was investigated with natural seawater (100% NSW) and diluted natural seawater (10% NSW). Filament growth and branching patterns of young parthenosporophytes were observed at 7, 10 and 15 days after gamete release. The elongation of primary filaments was significantly faster when *E. subulatus* was cultivated in diluted natural seawater than in undiluted seawater (**Figures 1A, B**). Cell sizes were not affected by these treatments (**Table 1**), indicating that the number of cells rather than cell length triggered the difference observed on filament growth. After 15 days of cultivation in 10% NSW, the number of cells (15 cells) in the primary filaments (PFs) was nearly twice the number obtained in 100% NSW (8 cells, **Figure 1B**).

**Table 1:** Size parameters of cells and filaments of *E. subulatus* cultivated in seawater featuring distinct salinities.

	10% NSW	100% NSW
Length of apical cells (µm)	35 ± 12	32 ± 8
Diameter of apical cells (µm)	7 ± 1	7 ± 1
Length of round cells (µm)	19 ± 4	15 ± 6
Diameter of round cells (µm)	11 ± 2	9 ± 3
Total length of PF made of ~4 cells (µm)	116 ± 15	111 ± 15

The number of secondary filaments (SFs) is a function of the size of the PF. To allow comparisons between individuals, the number of SFs was scored for PFs made of 6 to 8 cells. The number of SFs was significantly reduced when *E. subulatus* was cultivated in 10% NSW (56% of individuals featuring at least one SF, n=43) as compared to 100% NSW (96% of individuals featuring at least one SF, n=52) (**Figure 1C**). Observations were also performed for PFs made of 3 to 5 cells (n=95) and they led to a similar conclusion (**Supplementary Figure S4A**). These results indicate that the salinity of the culture medium impacts the pattern of SF emergence in *E. subulatus* (**Supplementary Figure S4B**), with a higher branching activity when the strain was cultivated at 100% NSW compared to 10% NSW.

**Figure 2:**
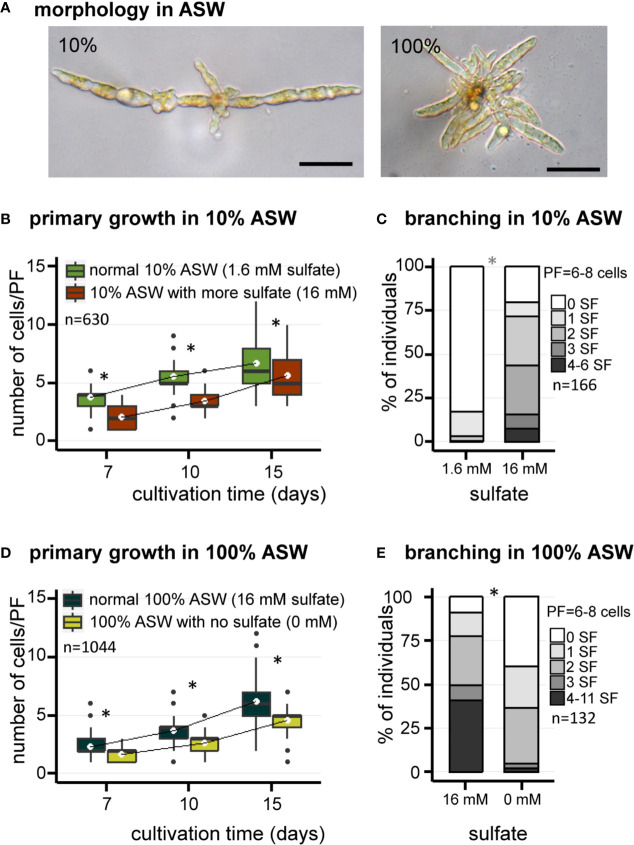
Morphology of *E. subulatus* grown in artificial seawater (ASW) featuring distinct salinities and sulfate contents. **(A)** Morphology of *E. subulatus* parthenosporphytes cultivated for 15 days in ASW featuring different salinities. Scale bar = 50 µm. **(B, D)** Elongation of *E. subulatus* primary filament (PF) was significantly greater (Mann-Whitney test: p < 0.001) when the gametes were cultivated in diluted seawater (10%) rather than in undiluted seawater (100%), except at later stages of development. **(C, E)** The branching activity of *E. subulatus* was significantly greater (Mann-Whitney test: p < 0.001) when the gametes were cultivated in undiluted seawater (100%) rather than in diluted seawater (10%). **(B)** Elongation of *E. subulatus* primary filament (PF) was not increased when the 10% ASW medium (containing 1.6 mM sulfate) was complemented with additional sulfate (i.e., up to 16 mM, the concentration found in 100% ASW) but, instead, was significantly reduced (Mann-Whitney test: p < 0.001). **(C)** The branching activity of *E. subulatus* significantly increased (Mann-Whitney test: p < 0.001) when the gametes were cultivated in a 10% ASW medium featuring a greater sulfate concentration. **(D)** Elongation of *E. subulatus* primary filament (PF) was significantly reduced (Mann-Whitney test: p < 0.001) when the gametes were cultivated in ASW 100% without sulfate. **(E)** The branching activity of *E. subulatus* was significantly reduced (Mann-Whitney test: p < 0.001) when the gametes were cultivated in a sulfate-depleted medium. To prevent a deleterious effect of sulfate starvation on protein synthesis, methionine was used as replacement of exogeneous sulfate when depleted. * above bars indicates significant differences.

Similar observations were made using artificial seawater (ASW) (**Figure 2**). Growth of *E. subulatus* was not as fast in ASW compared to NSW. The elongation of PFs was significantly faster in 10% ASW as compared to 100% ASW at early stages of development only, to reach similar sizes at 15 days (**Figures 2A, B, D**). However, the number of SFs was significantly reduced at 10% ASW (18% of individuals featuring at least one SF, n=141) as compared to 100% ASW (91% of individuals featuring at least one SF, n=94) (**Figures 2A, C, E**). This result confirms the previous observations made in NSW of a greatest branching activity when the strain is cultivated at normal seawater salinities.

### Presence of Sulfate in the Culture Medium Promotes Branching in *E. subulatus*

In order to study the contribution of sulfate in the responses registered above, the early morphogenesis of *E. subulatus* was observed with different sulfate contents in the medium. The chemical composition of the medium was controlled using ASW. To prevent a deleterious effect of sulfate starvation on protein synthesis, methionine was used as a replacement of exogenous sulfate when depleted.

The impact of sulfate depletion on the early development of *E. subulatus* was observed in artificial seawater at normal salinity (**Figure 2D**), a condition in which the branching activity was high (**Figure 2E**). The elongation of PFs was slightly reduced in a sulfate-depleted medium as compared to normal seawater (**Figure 2D**). The strongest impact was observed on the number of SFs, which was significantly reduced in the sulfate-depleted medium (61% of individuals featuring at least one SF, n=38) as compared to normal seawater (91% of individuals featuring at least one SF, n=94) (**Figure 2E**).

As previously shown, branching activity was reduced in diluted (10%) artificial seawater (**Figures 2C, E**). Complementation with 16 mM sulfate (the concentration found in undiluted ASW) did not improve the growth of primary filaments (**Figure 2B**). However *E. subulatus* recovered a branching activity similar to the one observed when cultivated in undiluted seawater (**Figures 2C, E**): 80% of individuals featured at least one SF upon sulfate addition (n=25), as compared to 18% only in 10% ASW (n=141).

### Sulfated Fucans Preferentially Localize in the Extracellular Matrix of Apical Cells

Previous immunolabeling experiments in another *E. subulatus*, strain Ec371 (accession CCAP 1310/196, origin Hopkins River Falls, Victoria, Australia) had indicated a positive correlation between the presence of highly sulfated fucans in cell walls (as detected by the BAM4 monoclonal antibody) and the salinity level (Torode et al., 2015). To further investigate this hypothesis, immunolabeling experiments were performed with *E. subulatus*, strain Bft15b, upon cultivation at different salinities and sulfate contents.

**Figure 3:**
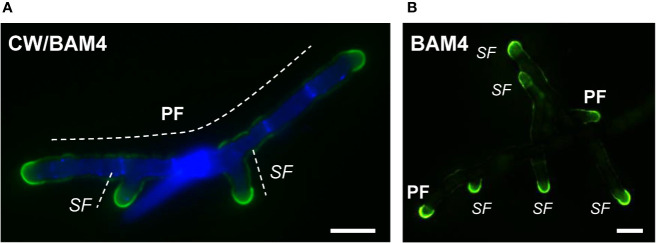
Immunofluorescence detection of the BAM4-fucan epitopes in *E. subulatus* filaments cultivated in 10% NSW. Micrographs showing filaments after 7 days **(A)** and 15 days **(B)** of development, respectively. The BAM4-binding (green fluorescence) is preferentially located at the apical cells. Cellulose was detected by Calcofluor White (blue fluorescence) in all cells expect at the tips. Micrograph in **(A)** shows the overlaid picture of the signals gained from the two channels, while **(B)** shows the green fluorescence only. PF = primary filament, SF = secondary filament. Scale bar = 20 µm.

**Figure 4:**
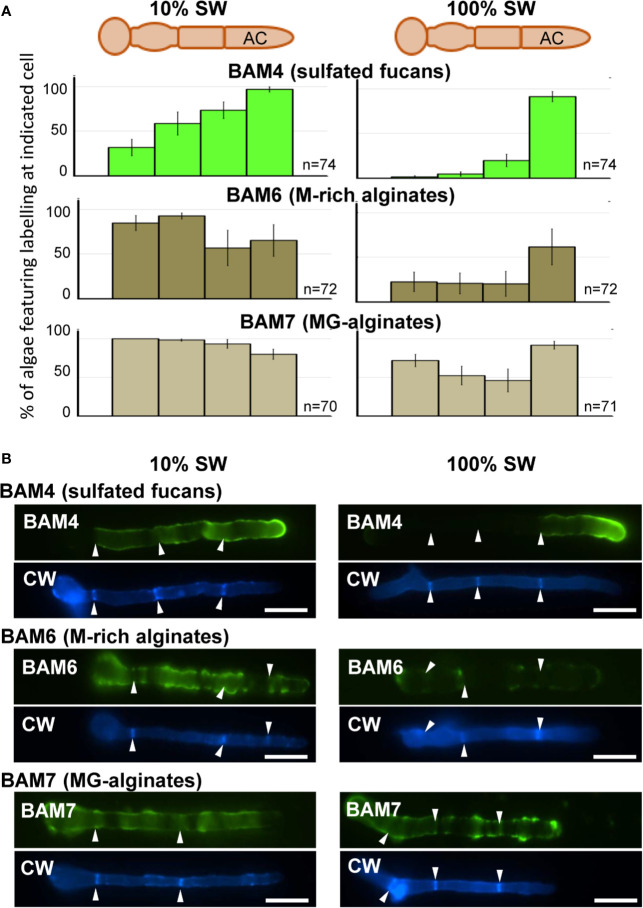
Immunofluorescence detection of fucan and alginate epitopes in *E. subulatus* cultivated in seawater featuring distinct salinities. **(A)** Histograms showing the proportion of individuals showing BAM4 labeling at the indicated cells, as a function of the surrounding salinity. Apical cells were always strongly labeled. Scores for alginate detection are shown for comparison with BAM6 detecting M-rich alginates and BAM7 detecting MG-alginates. Compared to BAM4-fucan they indicate a broader occurrence of those epitopes along the filaments. **(B)** Representative micrographs of individuals scored in (A) with the detection of fucan and alginate epitopes by the BAM antibodies (green fluorescence) and cellulose detection by Calcofluor White (blue fluorescence). Scale bar = 20 µm.

**Figure 5:**
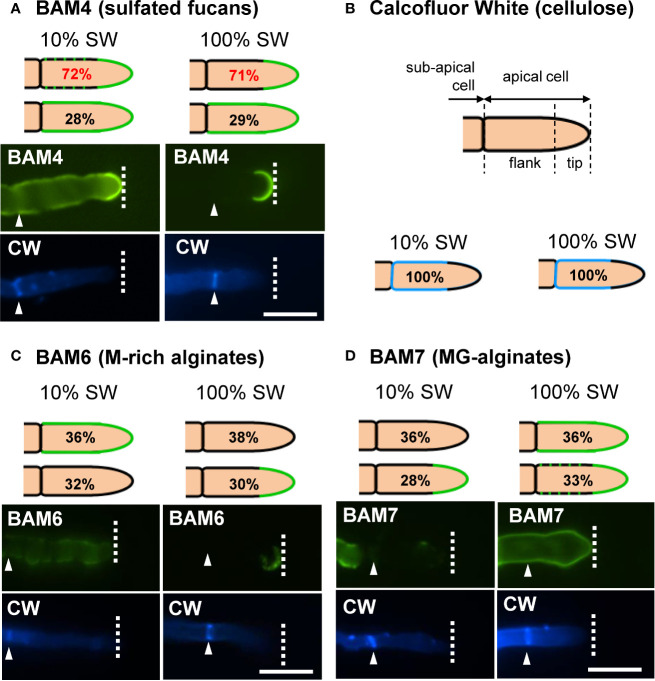
Immunofluorescence detection of fucan and alginate epitopes in *E. subulatus* apical cells. The schematic representations indicating the percentages of the BAM-immunofluorescence distribution in apical cells observed in 10% ASW and 100% ASW are shown together with representative pictures featuring labeling. Detections by the BAM antibodies (green fluorescence) and Calcofluor White (blue fluorescence) are shown. The dotted lines in the schematic representations indicates occasional labeling. The dotted line on the micrographs indicates the distal limit of the apical cell. **(A)** The BAM4 binding was preferentially located at the tip in either 10% or 100% ASW, but detection was also frequently observed at the flanks in 10% ASW but not in 100% ASW. The tip was always labeled by the BAM4 antibody. **(B)** Cellulose was detected by Calcofluor White in all walls except at the tip. Alginates epitopes as detected by the BAM6 **(C)** and the BAM7 **(D)** antibodies showed a greater variety of locations in apical cell walls than BAM4-fucans. n ≥ 33 for each culture condition and labeling type. Scale bar = 20 µm.

In natural seawater and irrespective of the salinity level, the BAM4 epitope was particularly abundant in the ECM of the apical cells of both the primary and secondary filaments (**Figure 3**). A BAM4 labeling was also observed along other cells, albeit with lower intensities as compared to the apical cell and with a decreasing occurrence from apical to distal cells within the filaments (n=148) (**Figure 4**). When the Bft15b strain was cultivated in undiluted seawater, the BAM4-apical labeling was more apparent, with almost no BAM4-detection from other cells (**Figure 4**). More precisely, at the apical cell level, the tip frequently featured a higher BAM4-labeling as compared to the whole cell (**Figure 5A**), a zone where the ECM is also devoid of cellulose (**Figure 5B**). The flanks were sometimes BAM4-labeled, and this was more apparent when the strain was cultivated at low salinity. Similar observations were made in the marine strain *Ectocarpus* sp. Ec32 (n=50) (**Supplementary Figure S5** and **Supplementary Figure S6**). We also investigated the occurrence of distinct alginate motifs using the BAM6 (M-rich alginates) and BAM7 (MG-alginates) antibodies in both *Ectocarpus* species (n=396). The alginate epitopes showed a more heterogeneous labeled pattern between cells or as a function of salinity (**Figures 4** and **5C, D**). In *Ectocarpus* sp. Ec32 strain, the BAM6 and BAM7 alginate epitopes showed a broader occurrence along the filaments (n=111) than previously stated (Rabillé et al., 2019a) (**Supplementary Figures S5** and **S6**). Overall, our results indicate that, in the early stages of both *Ectocarpus* species, the abundance of highly sulfated fucans at the tip of apical cells was a consistent feature.

### Incorporation of Sulfated Fucans in the Extracellular Matrix Depends on the Availability of Exogenous Sulfate

**Figure 6:**
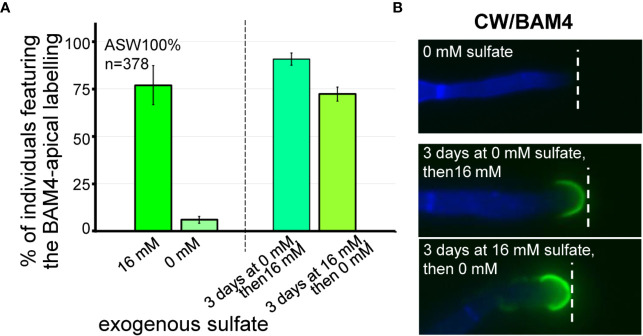
Impact of sulfate depletion on the BAM4-fucan detection in apical cells. All the *E. subulatus* filaments were observed after 15 days of culture in a medium based on 100% ASW but featuring different sulfate concentrations. **(A)** Histogram showing the proportion of labeled apical cells in the different culture conditions. The strong BAM4 labeling observed in normal ASW (16 mM sulfate) was not observed in a sulfate-depleted medium (0 mM sulfate) but it was recovered when the individuals were placed back in normal ASW after 3 days of culture without sulfate (i.e., 3 days at 0 mM sulfate followed by 12 days at 16 mM sulfate). Depleting the exogenous sulfate after 3 days of culture was of little impact on the BAM4 detection (i.e., 3 days at 16 mM sulfate followed by 12 days at 0 mM sulfate). **(B)** Micrographs showing the absence of BAM4-detection in apical cells of *E. subulatus* cultivated in a sulfate-deprived medium (0 mM sulfate) and its recovering upon sulfate addition after 3 days of culture (i.e., 3 days at 0 mM sulfate followed by 12 days at 16 mM sulfate). When *E. subulatus* was first grown in normal ASW prior to sulfate depletion (i.e., 3 days at 16 mM sulfate followed by 12 days at 0 mM sulfate), the BAM4 epitopes were still detected on apical cells.

The effect of the availability of exogenous sulfate on the occurrence of the BAM4 epitopes in the ECM was assessed by performing immunolabeling experiments on *E. subulatus* filaments cultivated in sulfate-depleted ASW. In normal artificial seawater, which contains 16 mM sulfate, 77% of the individuals (n=100) show a BAM4-labeling on apical cells. In contrast, after cultivation in sulfate-free ASW for 15 days, *Ectocarpus* apical cells showed almost no BAM4 binding (**Figure 6**, **Supplementary Figure S7**). Only in rare cases (6%, n=125) recognition by the monoclonal was apparent, and if so only at the tip. In order to assess the reversible effect of sulfate depletion on BAM4 recognition, the individuals were first grown in sulfate-free ASW for 3 days prior to addition of 16 mM sulfate and then further cultivated for 12 days. In this condition, the detection of the fucan epitopes was fully restored in apical cells, with 91% of *Ectocarpus* filaments (n=64) showing a BAM4-binding (**Figure 6**). Inversely, when *E. subulatus* was first grown for 3 days in normal ASW prior to sulfate depletion and additional cultivation for 12 days, the BAM4 epitopes were still detected on apical cells (72%, n=89).

## Discussion

### *Ectocarpus* Filaments Feature Different Patterns of Extracellular Matrix Polysaccharides Along the Growth Axis

The ECM of brown algae is made of crystalline or semi-crystalline elements, namely cellulose, other β-glucans and alginates, as well as more flexible elements, including fucose-containing sulfated polysaccharides (FCSPs) (Deniaud-Bouët et al., 2014; Deniaud-Bouët et al., 2017). Cell wall rigidity depends on the interlocking of two macromolecular networks, that of β-glucans and FCSPs on the one hand and that of alginates and cross-linking phenols on the other hand. The first macromolecular network is thought to be ancestral while the other was acquired more recently upon horizontal gene transfer (Deniaud-Bouët et al., 2014). Structural or enzymatic proteins such as arabinogalactan proteins and haloperoxidases and mannuronate-C5-epimerases respectively, are also involved with the mechanical properties of the extracellular matrix of brown algae (Fischl et al., 2016; Hervé et al., 2016).

Here we used monoclonal antibodies specific of either alginates (referred to as BAM6 and BAM7, Torode et al., 2016) or highly sulfated fucans (BAM4, Torode et al., 2015) to monitor the incorporation of these polysaccharides in the ECM during the early stages of the development of *Ectocarpus*. In *E. subulatus* Bft15b and *Ectocarpus* sp. Ec32, cellulose is detected in the ECMs along the filament, except at the tip. In contrast, highly sulfated fucans are abundant at the dome of the apical cells (**Figures 3**–**5**; **Supplementary Figures S5** and **S6**). Alginates are evenly distributed at the tips and in the flanks of apical cells of elongating filaments. Altogether, with the exception of cultures in diluted NSW and ASW, *Ectocarpus* apical cells, whether from primary or secondary filaments, all have a similar apparent cell wall composition, which differs from that of other cells. These results are similar to observations made in other brown algal models featuring apical growth, such as the *Fucus* zygotes (Torode et al., 2016).

### Presence of Sulfate Promotes Branching of *Ectocarpus* Filaments and Incorporation of Sulfated Fucans in the ECM of Apical Cells

Salinity of the culture medium was known to influence the architectural plasticity of *Ectocarpus*, with normal salinities promoting the emergence of secondary filaments (Dittami et al., 2012; Torode et al., 2015). We here confirm and quantify these observations, in both natural (**Figure 1**) and artificial (**Figure 2**) seawater.

Sulfate is a core element of many components required for both normal algal growth and, presumably, osmotic regulation. Applying a low salinity medium by diluting seawater impacts on the concentration of such constituents, including sulfate. Sulfur also is an essential element in many metabolic pathways. To prevent from sulfur starvation in sulfate-free seawater, methionine is added to the culture medium (Crayton et al., 1974). In artificial seawater complemented with methionine, depletion of sulfate markedly reduced the emergence of secondary filaments (**Figure 2E**), whereas upon cultivation with diluted ASW but containing 16 mM sulfate (i.e., the concentration found in undiluted ASW), branching activities were similar to those observed with undiluted, normal seawater (**Figure 2C**). It follows that it is not the salinity per se which promotes branching but the presence of sulfate in the culture medium.

This is correlated with the incorporation of sulfated fucans at the tip of apical cells of the primary and secondary *Ectocarpus* filaments in cultures in ASW containing 16 mM sulfate, as opposed to cultures in sulfate-free ASW, which did not feature any BAM4 labeling (**Figure 6**). This indicates that the incorporation of the BAM4 epitopes at the dome of apical cells is directly linked with exogenous supply of sulfate in the culture medium. However, when cultures were initiated in sulfate-containing ASW for 3 days, then transferred in sulfate-free ASW, the BAM4 labeling was maintained, indicating that the pool of sulfate built after 3 days in *Ectocarpus* filaments was sufficient to sustain for more than a week the incorporation of sulfated fucans at the dome of apical cells (**Figure 6A**).

### The Role of Highly Sulfated Fucans in Tip Growth and Branching

In land plants, the loosening and constant remodeling of the cell walls material at the tip has been shown to be a key mechanism facilitating tip growth (Parre and Geitmann, 2005; Chebli et al., 2012). In contrast to plants, in *Ectocarpus* apical cells the tip is stiffer than the shanks (Rabillé et al., 2019b). The cell wall being essentially made of alginates and FCSPs, the question arises on which components contributes to strengthen the wall at the dome and prevent tip bursting. The dome is known to be devoid of cellulose in *Ectocarpus* (Le Bail et al., 2008; **Supplementary Figure S3**). Rabillé et al. (2019a) have suggested that alginates may contribute to mechanical reinforcement. However, in our hands, the specific location of alginates at the dome was not apparent, indicating a more complex scheme. While the BAM4 epitope is particularly abundantly at the tip, the mechanical properties of fucans would unlikely support the building of stiff walls, unless they are cross-linked with other components. Insoluble mixed linkage glucans have recently been detected in brown algal cell walls, including from the *Ectocarpus* sp. Ec32 strain (Salmeán et al., 2017). An initial screening indicates their positioning at the tip of apical growing cells, although in rare occasions and with low fluorescence intensities (**Supplementary Figure S8**). The screening of additional glucan epitopes (e.g., xyloglucan, callose) failed to retrieve signals (**Supplementary Figure S8**). A more detailed investigation of components location in cells walls of apical cells, including glycans and phlorotannins may extend our understanding of their roles and possible interactions in wall remodeling in tip-growing cells of brown algae.

In any case, it altogether appears that in *Ectocarpus* filaments, there is a remarkable correlation between the availability of exogeneous sulfate in the culture medium, the presence of sulfated fucans at the dome of apical cells, as well as tip growth and branching activity. The question then arises of which are mechanism(s) at the basis of the function of sulfated fucans in tip growth and the emergence of SFs. FCSPs are highly hygroscopic macromolecules, a property linked to their high density of anionic charges (Kloareg et al., 1986). They also are highly flexible polysaccharides. Both of these intrinsic properties would contribute to loosen the ECM network, providing more moisture and elasticity. Combination of turgor pressure with localized cell wall weakening would then give rise to elongation of apical cells and to the emergence of SFs.

In *Fucus* developing embryos, highly sulfated fucans are deposited at the rhizoid tip during the emergence and the elongation of the rhizoid (Quatrano et al., 1991; Torode et al., 2016). They are considered a source of position-dependent information required for cell polarization (Kropf et al., 1988; Berger et al., 1994; Bisgrove and Kropf, 2001). In particular, highly sulfated fucans are assumed to be involved in an axis-stabilizing complex (ASC), located at the rhizoid apex, which physically bridges the ECM to the cytoskeleton through trans-membrane proteins such as those found in the focal contact of animal cells, e.g., integrin and vitronectin (Kropf et al., 1988; Quatrano et al., 1991; Wagner et al., 1992). Polar growth is then sustained through cytosolic gradients of H^+^ and Ca^2+^ (Berger and Brownlee, 1993; Taylor et al., 1996). Similar mechanisms are likely to occur in the early development of *Ectocarpus* filaments.

In conclusion, in *Ectocarpus*, presence of exogenous inorganic sulfate is required for the incorporation of sulfated fucans in the ECM of apical cells, leading to the anisotropic growth of the primary filaments and to the emergence of new filaments. We propose that the visco-elastic properties of sulfated fucans play a major role in the initiation of this developmental program. These observations provide a cell biology basis to investigate the genetics of the function of extracellular matrix polysaccharides in polarization and tip growth in the model brown alga *Ectocarpus*.

## Data Availability Statement

The original contributions presented in the study are included in the article/supplementary material; further inquiries can be directed to the corresponding author.

## Author Contributions

CH designed the research. AS performed the experiments. CH and AS analyzed the data. CH and BK wrote the manuscript. SK was involved in performing experiments.

## Funding

AS received a grant from the Brittany region (ARED_8979 ECTOPAR). CH acknowledges funding from the French National Research Agency with regard to the investment expenditure program Idealg (http://www.idealg.ueb.eu/, grant agreement no. ANR-10-BTBR-04).

## Conflict of Interest

The authors declare that the research was conducted in the absence of any commercial or financial relationships that could be construed as a potential conflict of interest.
