# Supplementary material for: Presence of Exogenous Sulfate Is Mandatory for Tip Growth in the Brown Alga Ectocarpus subulatus
Source: Front Plant Sci. 2020 Aug 18;11:1277. doi: 10.3389/fpls.2020.01277 (PMC7461865; doi:10.3389/fpls.2020.01277)
Supplement: Supplementary file 1 [file DataSheet_1.pdf]

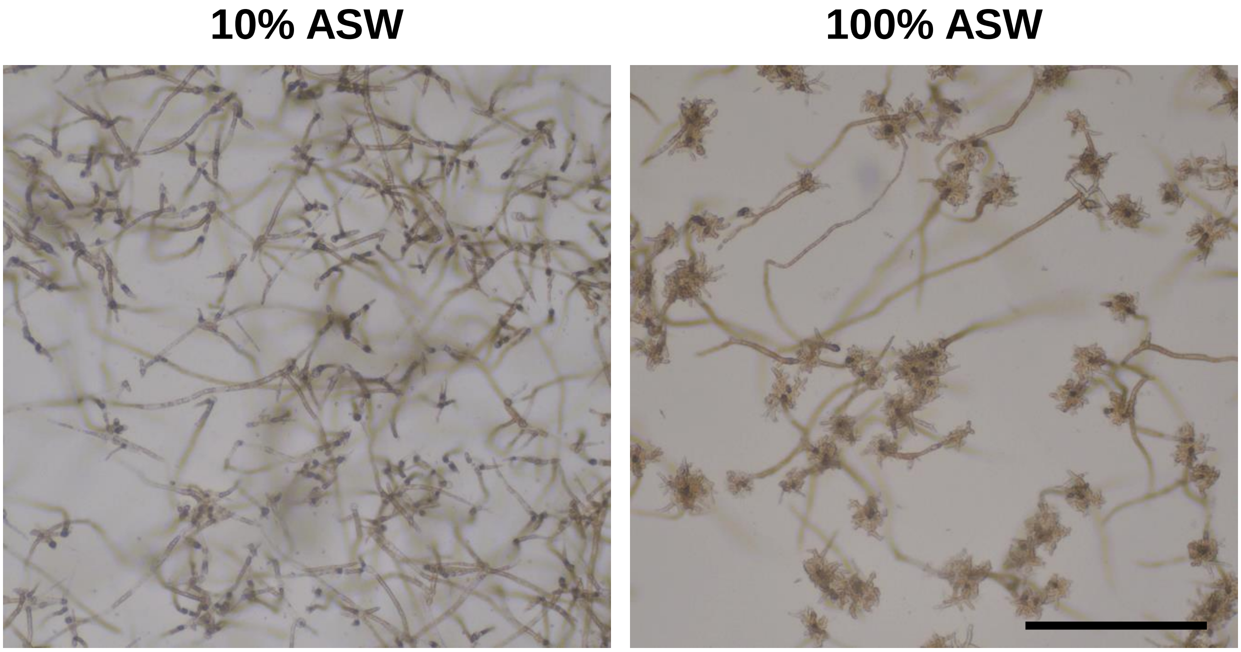

Supplementary Figure S1. Morphology of parthenosporophytes from the *E. subulatus* Bft15b strain, cultivated five weeks in ASW featuring distinct salinities. Scale bar = 0.5 mm. Long filaments are observed at low salinity (10% ASW), compared to star-shaped thalli in normal seawater (100% ASW), this later morphotype being similar to the marine strain *Ectocarpus* sp. Ec32.

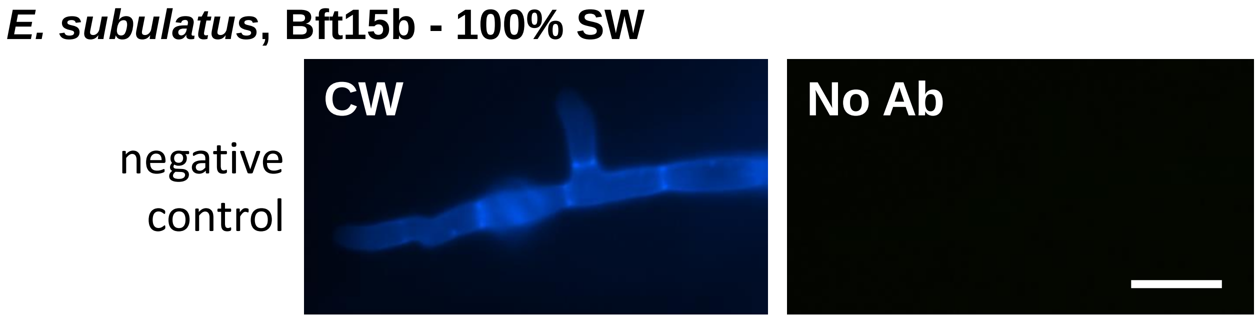

Supplementary Figure S2. Negative control micrograph of *E. subulatus* labelled without primary antibody. The Calcofluor White staining is shown to value all cell walls. Scale bar = 20 µm

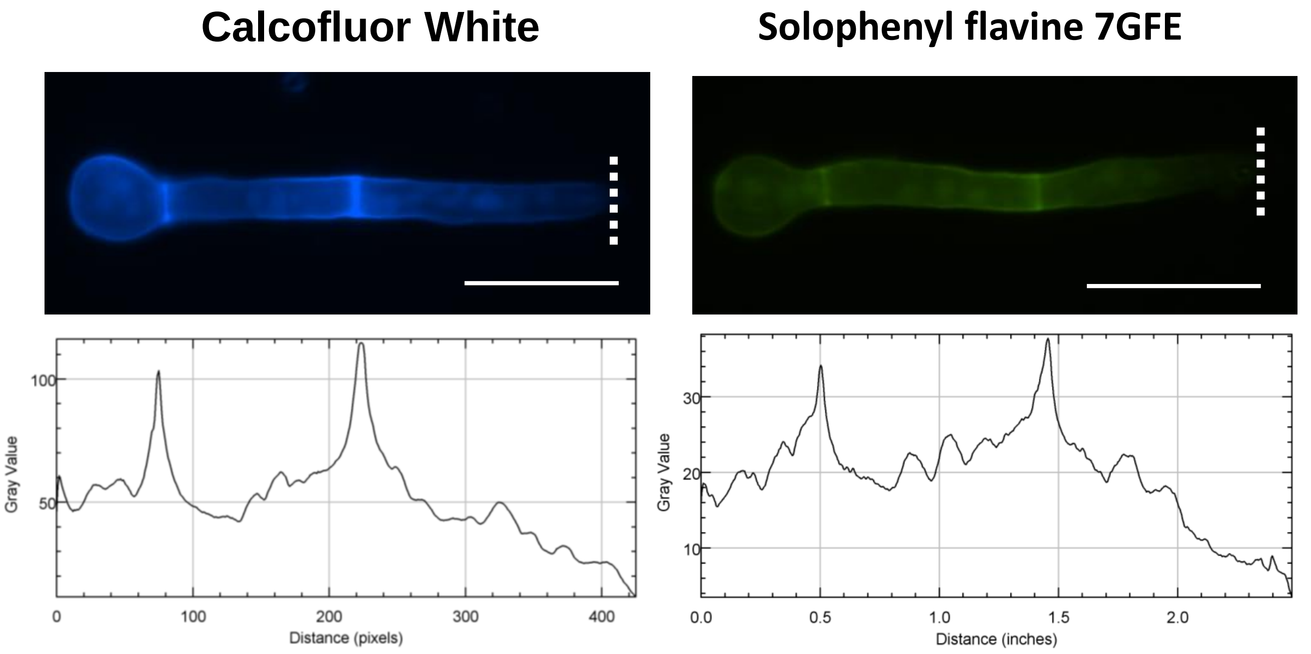

Supplementary Figure S3. Cellulose detection in *Ectocarpus* sp. Ec32 strain using two distinct stains. Calcofluor White (blue fluorescence) stains β-glucans including cellulose. Solophenly flavine 7GFE (green fluorescence) stains β-1,4-glucans including cellulose and xyloglucan (the later not known to occur in brown algae). The plots below the micrographs indicate the relative value of the signal along the filaments with the corresponding stain. The two stains show a similar pattern of detection and Calcofluor White was later used as the main stain for detecting cellulose in these cells. In all cases tips were devoid of cellulose. The dotted line indicates the distal limit of the apical cell. Scale bar = 20 µm.

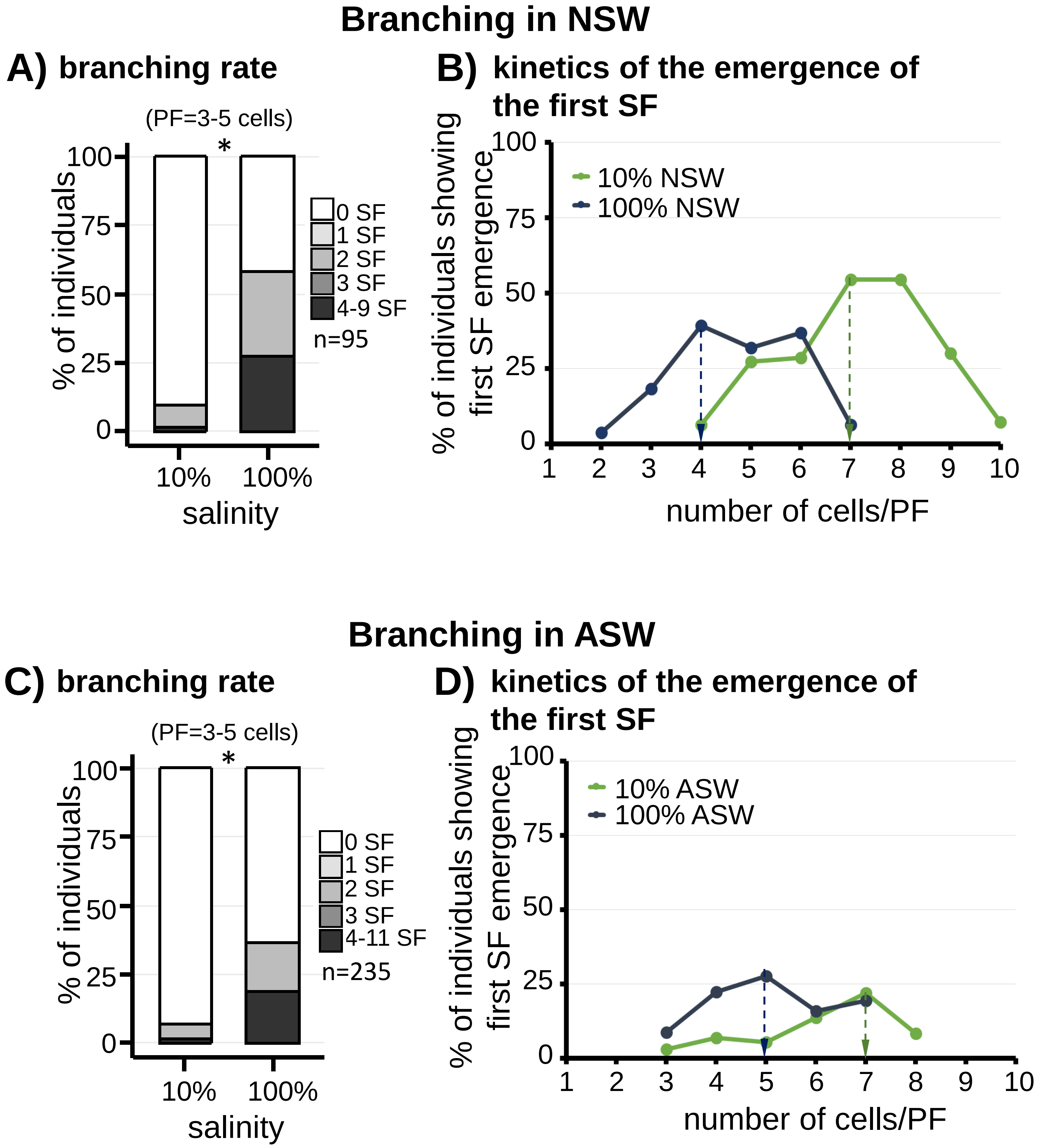

Supplementary Figure S4. Morphology of *E. subulatus* in seawater featuring distinct salinities. (A, B) Branching in natural seawater. (C, D) Branching in artificial seawater. (A, C) The branching activity of *E. subulatus* was greater when gametes were cultivated in undiluted seawater (100%) rather than in diluted seawater (10%). Cases of primary filaments of 3 to 5 cells are shown. Asterisks indicate a significant difference between culture conditions (Mann-Whitney test: $p<0.001$). **(B, D)** Another illustration of this is the kinetics of branching. The first secondary filament (SF) always emerged earlier (i.e. on shorter primary filaments) in undiluted seawater (100%) as compared to cultures in diluted seawater (10%). This observation also applied for the subsequent secondary filaments (data not shown).

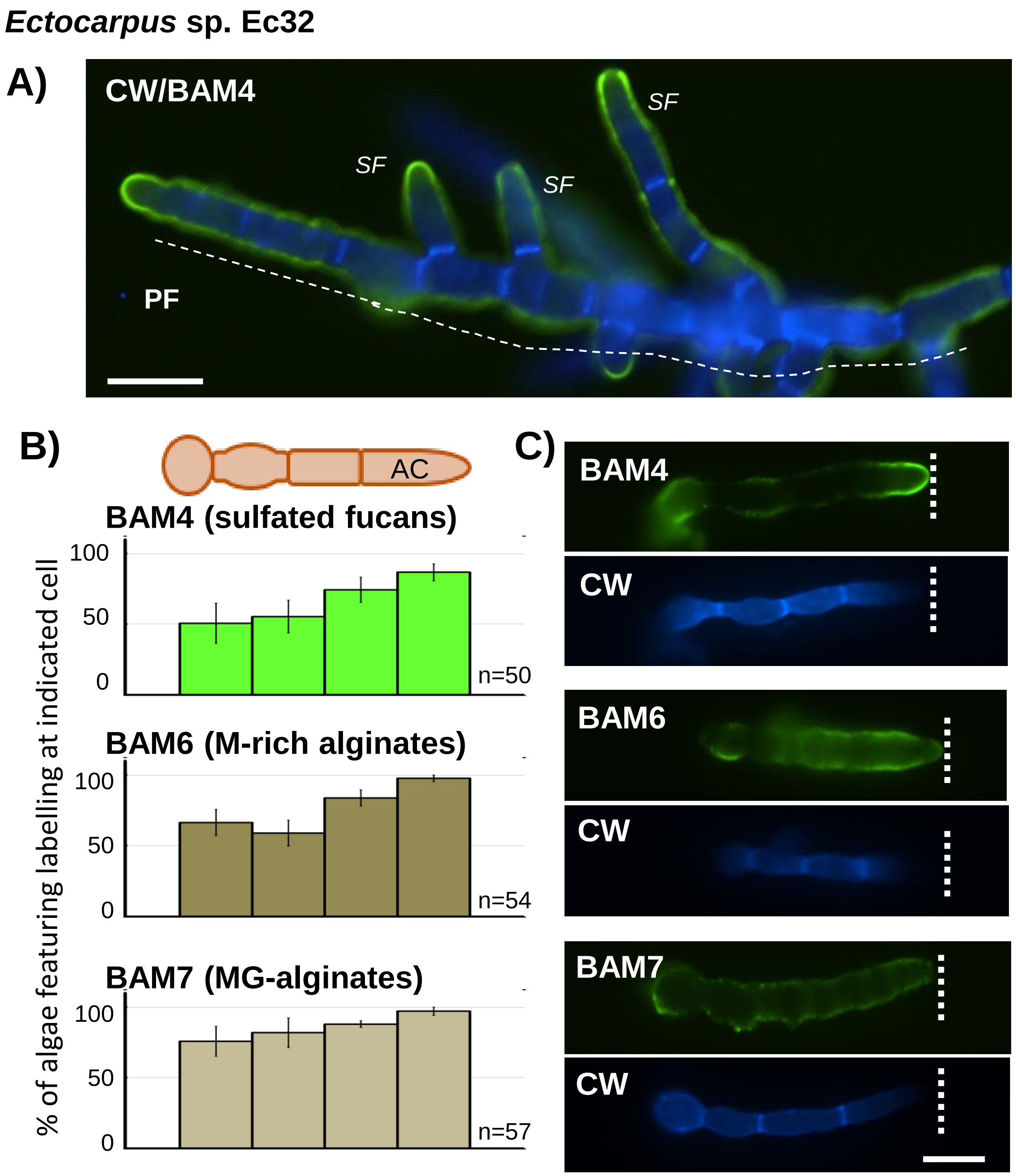

Supplementary Figure S5. Immunofluorescence detection of cell-wall epitopes in *Ectocarpus* sp. Ec32 strain cultivated in natural seawater. (A) Micrographs showing a 20 day-filament. The BAM4-binding (green fluorescence) was preferentially located at the apical cells. Cellulose was detected by Calcofluor White (blue fluorescence) in all cells expect at the tips. PF = primary filament, SF = secondary filament. Scale bar = 20 µm. **(B)** Histograms showing the proportion of individuals showing a labelling at the indicated cells and as a function of the salinity. Observations were made for sulfated fucans (BAM4), distinct alginates motifs (BAM6, BAM7) and cellulose (histogram not shown: all cells being always labelled except at the tips). The BAM4 labelling was always stronger in apical cells. **(C)** Representative micrographs of individuals scored in (B) with the detection of fucan and alginate epitopes by the BAM antibodies (green fluorescence) and cellulose detection by Calcofluor White (blue fluorescence). The dotted line indicates the distal limit of the apical cell. Scale bar = 20 µm.

Ectocarpus sp. Ec32

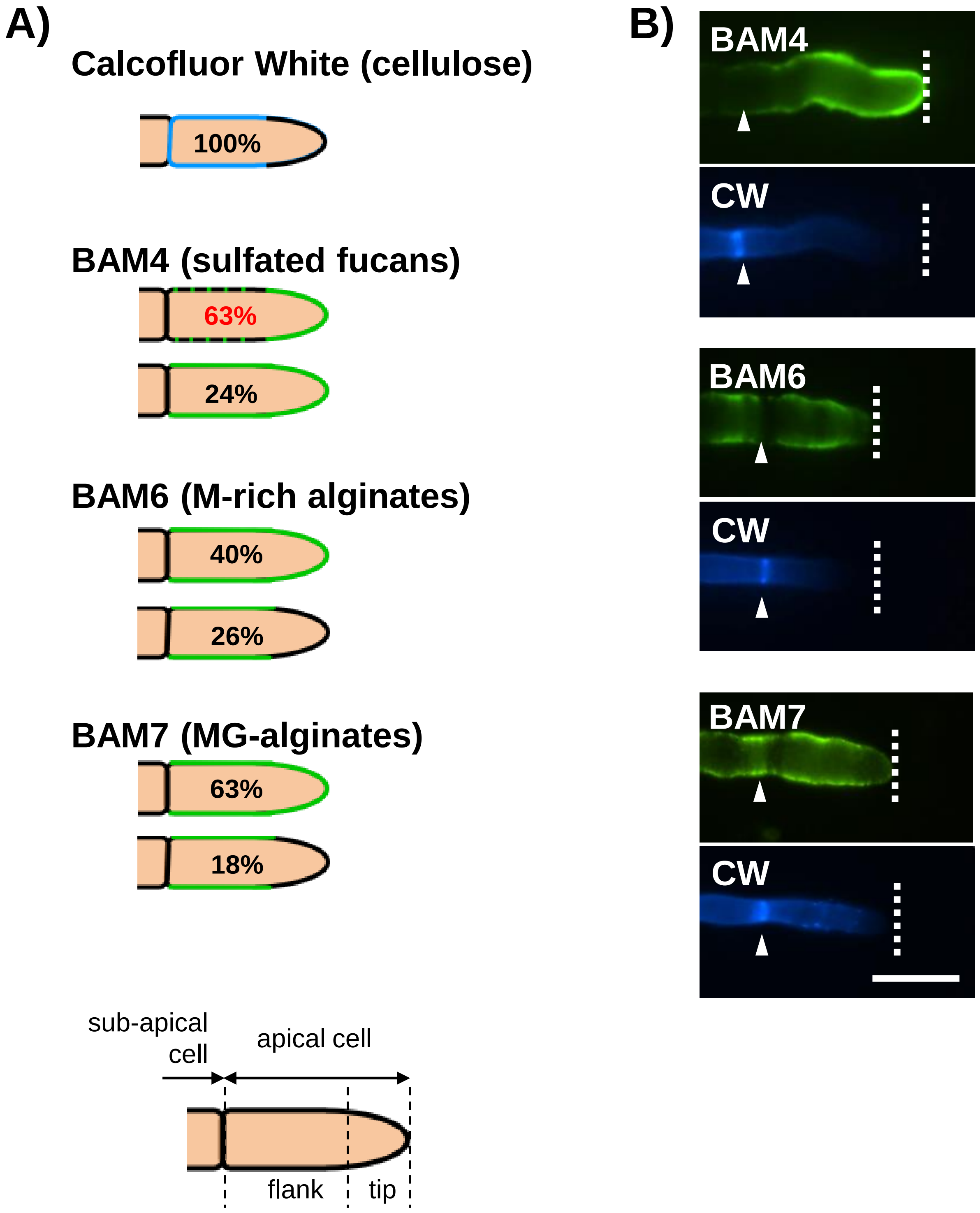

Supplementary Figure S6. Immunofluorescence detection of cell-wall epitopes in the apical cells of the *Ectocarpus* sp. Ec32 strain cultivated in natural seawater. (A) Schematic representation indicating the proportion of the immunofluorescence distributions observed in apical cells. Observations were made for sulfated fucans (BAM4), distinct alginates motifs (BAM6, BAM7) and cellulose (Calcofluor White). The dotted line indicates occasional labelling. The BAM4 labelling was always stronger at the tip of apical cells. **(B)** Representative micrographs of individuals scored in (B) with the detection of fucan and alginate epitopes by the BAM antibodies (green fluorescence) and cellulose detection by Calcofluor White (blue fluorescence). The dotted line indicates the distal limit of the apical cell. Scale bar = 20 µm.

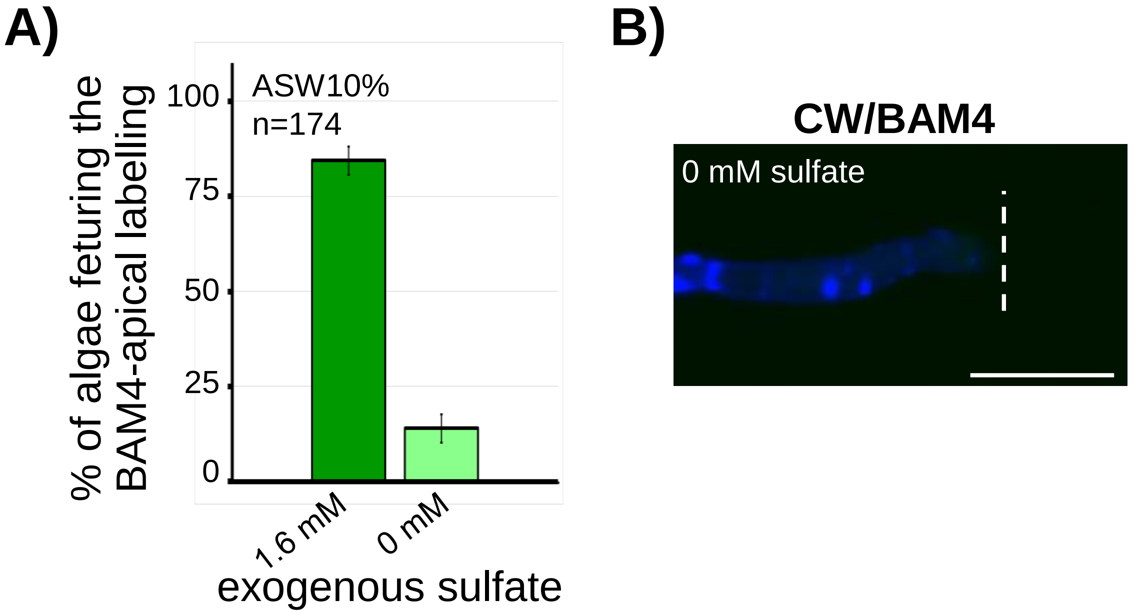

Supplementary Figure S7. Impact of a sulfate depletion on the BAM4-fucan detection in apical cells. All the *E. subulatus* filaments were observed after 15 days of culture. **(A)** Histogram showing the proportion of labelled apical cells in the various culture conditions. The strong BAM4 labelling observed in 10% ASW (containing 1.6 mM sulfate) was not observed in a sulfate-depleted medium (0 mM sulfate). **(B)** Micrographs showing the absence of a BAM4-detection in apical cells of *E. subulatus* cultivated in a sulfate-deprived medium. Note that the reverse experiments made at 100% ASW, as shown in Figure 6, were not scored at 10% ASW, as the *E. subulatus* cultures have a much reduced growth rate at 10% ASW and are too delicate to cope with medium changes. The dotted line indicates the distal limit of the apical cell. Scale bar = 20 µm.

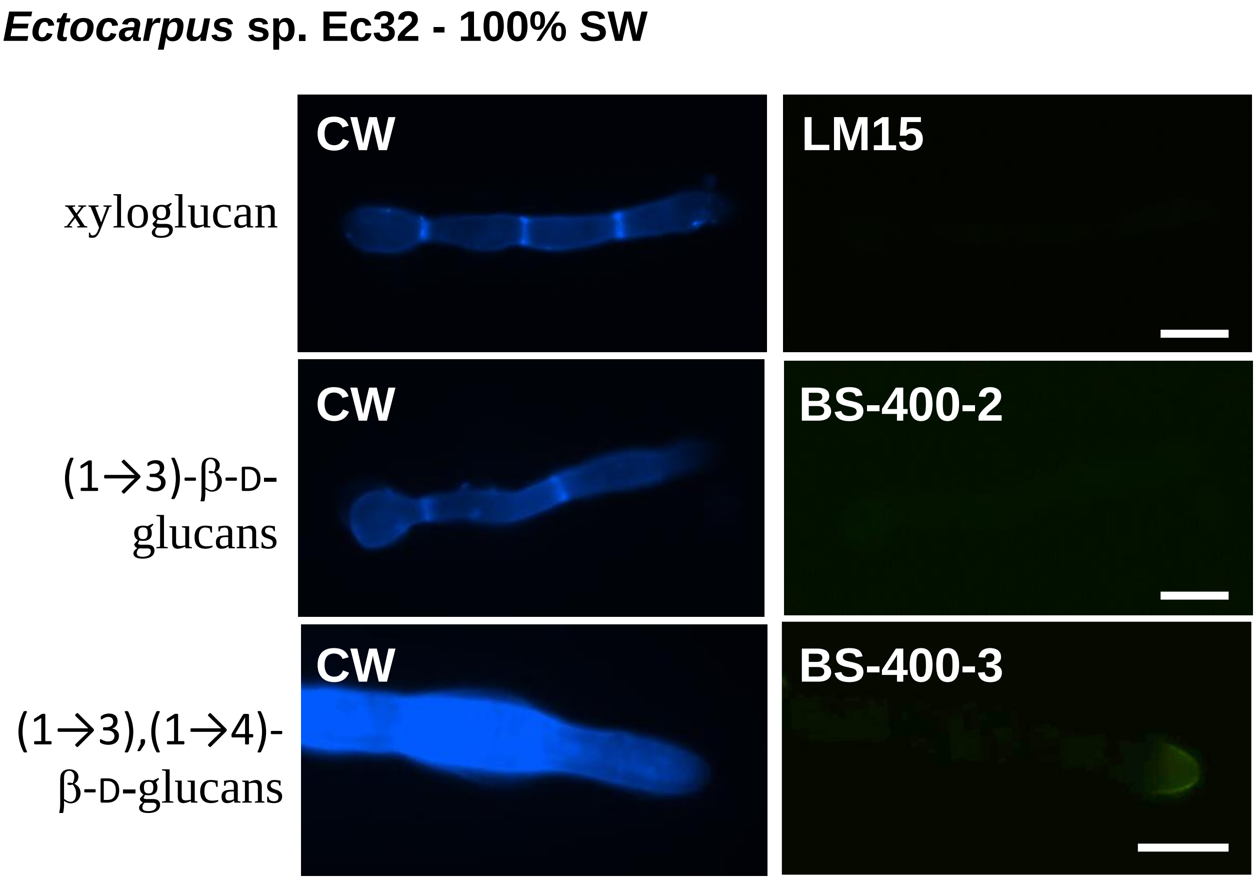

Supplementary Figure S8. Immunofluorescence screening of plant cell-wall epitopes in the apical cells of *Ectocarpus* sp. Ec32 strain cultivated in normal seawater. Xyloglucan and (1→3)-β-D-glucans were not detected in the *Ectocarpus* filaments, using the LM15 and BS-000-2 antibodies respectively. Some faint labelling with the BS-400-3 antibody was observed in some rare cases at the tips and this may indicate the deposition of mixed linkage glucans ((1→3),(1→4)-β-D-glucans). Scale bars = 20 µm.
